# Supplementary material for: Disaggregating proportional multistate lifetables by population heterogeneity to estimate intervention impacts on inequalities
Source: Popul Health Metr. 2022 Jan 15;20:6. doi: 10.1186/s12963-022-00282-7 (PMC8761347; doi:10.1186/s12963-022-00282-7)
Supplement: Supplementary file 1 — Additional file 1. Supplementary Material. [file 12963_2022_282_MOESM1_ESM.docx]

# Appendix 2: Disaggregation input parameters

Supplementary Table 1: Base Mortality rate and Morbidity Ratios for Māori Females

| **Ethnicity** | **Sex** | **Age** | **Mortality rate** | **Morbidity Ratio** |
| --- | --- | --- | --- | --- |
| Māori | F | 0 | 0.005244 | 0.016838 |
| Māori | F | 1 | 0.00052 | 0.032138 |
| Māori | F | 2 | 0.00015 | 0.032138 |
| Māori | F | 3 | 0.00013 | 0.032138 |
| Māori | F | 4 | 0.00011 | 0.032138 |
| Māori | F | 5 | 0.0001 | 0.035087 |
| Māori | F | 6 | 8.00E-05 | 0.035087 |
| Māori | F | 7 | 6.00E-05 | 0.035087 |
| Māori | F | 8 | 6.00E-05 | 0.035087 |
| Māori | F | 9 | 7.00E-05 | 0.035087 |
| Māori | F | 10 | 0.0001 | 0.036172 |
| Māori | F | 11 | 0.00015 | 0.036172 |
| Māori | F | 12 | 0.00022 | 0.036172 |
| Māori | F | 13 | 0.00032 | 0.036172 |
| Māori | F | 14 | 0.00041 | 0.036172 |
| Māori | F | 15 | 0.0005 | 0.101246 |
| Māori | F | 16 | 0.00058 | 0.101246 |
| Māori | F | 17 | 0.00063 | 0.101246 |
| Māori | F | 18 | 0.00065 | 0.101246 |
| Māori | F | 19 | 0.00066 | 0.101246 |
| Māori | F | 20 | 0.00065 | 0.126133 |
| Māori | F | 21 | 0.00064 | 0.126133 |
| Māori | F | 22 | 0.00062 | 0.126133 |
| Māori | F | 23 | 0.0006 | 0.126133 |
| Māori | F | 24 | 0.00059 | 0.126133 |
| Māori | F | 25 | 0.00059 | 0.146245 |
| Māori | F | 26 | 0.0006 | 0.146245 |
| Māori | F | 27 | 0.00064 | 0.146245 |
| Māori | F | 28 | 0.00068 | 0.146245 |
| Māori | F | 29 | 0.00074 | 0.146245 |
| Māori | F | 30 | 0.0008 | 0.150982 |
| Māori | F | 31 | 0.00085 | 0.150982 |
| Māori | F | 32 | 0.00089 | 0.150982 |
| Māori | F | 33 | 0.00093 | 0.150982 |
| Māori | F | 34 | 0.00097 | 0.150982 |
| Māori | F | 35 | 0.001011 | 0.157912 |
| Māori | F | 36 | 0.001061 | 0.157912 |
| Māori | F | 37 | 0.001121 | 0.157912 |
| Māori | F | 38 | 0.001201 | 0.157912 |
| Māori | F | 39 | 0.001311 | 0.157912 |
| Māori | F | 40 | 0.001441 | 0.160344 |
| Māori | F | 41 | 0.001611 | 0.160344 |
| Māori | F | 42 | 0.001822 | 0.160344 |
| Māori | F | 43 | 0.002062 | 0.160344 |
| Māori | F | 44 | 0.002333 | 0.160344 |
| Māori | F | 45 | 0.002643 | 0.18003 |
| Māori | F | 46 | 0.002974 | 0.18003 |
| Māori | F | 47 | 0.003346 | 0.18003 |
| Māori | F | 48 | 0.003737 | 0.18003 |
| Māori | F | 49 | 0.004149 | 0.18003 |
| Māori | F | 50 | 0.00458 | 0.17632 |
| Māori | F | 51 | 0.005033 | 0.17632 |
| Māori | F | 52 | 0.005495 | 0.17632 |
| Māori | F | 53 | 0.005998 | 0.17632 |
| Māori | F | 54 | 0.006541 | 0.17632 |
| Māori | F | 55 | 0.007156 | 0.196225 |
| Māori | F | 56 | 0.007841 | 0.196225 |
| Māori | F | 57 | 0.008617 | 0.196225 |
| Māori | F | 58 | 0.009495 | 0.196225 |
| Māori | F | 59 | 0.010495 | 0.196225 |
| Māori | F | 60 | 0.011637 | 0.228255 |
| Māori | F | 61 | 0.012923 | 0.228255 |
| Māori | F | 62 | 0.014373 | 0.228255 |
| Māori | F | 63 | 0.015977 | 0.228255 |
| Māori | F | 64 | 0.017696 | 0.228255 |
| Māori | F | 65 | 0.01954 | 0.261278 |
| Māori | F | 66 | 0.021459 | 0.261278 |
| Māori | F | 67 | 0.023463 | 0.261278 |
| Māori | F | 68 | 0.025513 | 0.261278 |
| Māori | F | 69 | 0.027608 | 0.261278 |
| Māori | F | 70 | 0.029707 | 0.284812 |
| Māori | F | 71 | 0.031811 | 0.284812 |
| Māori | F | 72 | 0.033919 | 0.284812 |
| Māori | F | 73 | 0.036125 | 0.284812 |
| Māori | F | 74 | 0.038543 | 0.284812 |
| Māori | F | 75 | 0.041312 | 0.321474 |
| Māori | F | 76 | 0.044548 | 0.321474 |
| Māori | F | 77 | 0.048371 | 0.321474 |
| Māori | F | 78 | 0.052895 | 0.321474 |
| Māori | F | 79 | 0.058254 | 0.321474 |
| Māori | F | 80 | 0.06456 | 0.375736 |
| Māori | F | 81 | 0.071947 | 0.375736 |
| Māori | F | 82 | 0.080538 | 0.375736 |
| Māori | F | 83 | 0.090373 | 0.375736 |
| Māori | F | 84 | 0.099312 | 0.375736 |
| Māori | F | 85 | 0.109614 | 0.477049 |
| Māori | F | 86 | 0.121253 | 0.477049 |
| Māori | F | 87 | 0.134206 | 0.477049 |
| Māori | F | 88 | 0.148442 | 0.477049 |
| Māori | F | 89 | 0.164038 | 0.477049 |
| Māori | F | 90 | 0.181306 | 0.477049 |
| Māori | F | 91 | 0.200661 | 0.477049 |
| Māori | F | 92 | 0.222531 | 0.477049 |
| Māori | F | 93 | 0.247347 | 0.477049 |
| Māori | F | 94 | 0.275385 | 0.477049 |
| Māori | F | 95 | 0.306023 | 0.477049 |
| Māori | F | 96 | 0.339312 | 0.477049 |
| Māori | F | 97 | 0.375319 | 0.477049 |
| Māori | F | 98 | 0.414032 | 0.477049 |
| Māori | F | 99 | 0.455407 | 0.477049 |
| Māori | F | 100 | 0.499309 | 0.477049 |
| Māori | F | 101 | 0.545572 | 0.477049 |
| Māori | F | 102 | 0.593899 | 0.477049 |
| Māori | F | 103 | 0.6439 | 0.477049 |
| Māori | F | 104 | 0.695029 | 0.477049 |
| Māori | F | 105 | 0.746632 | 0.477049 |
| Māori | F | 106 | 0.746632 | 0.477049 |
| Māori | F | 107 | 0.746632 | 0.477049 |
| Māori | F | 108 | 0.746632 | 0.477049 |
| Māori | F | 109 | 0.746632 | 0.477049 |
| Māori | F | 110 | 0.746632 | 0.477049 |

Supplementary Table 2: Table Initial Population Counts for Māori Females

| **Ethnicity** | **Sex** | **Age** | **Population count** |
| --- | --- | --- | --- |
| Māori | F | 0-4 | 44100 |
| Māori | F | 5-9 | 34700 |
| Māori | F | 10-14 | 33800 |
| Māori | F | 15-19 | 32600 |
| Māori | F | 20-24 | 30100 |
| Māori | F | 25-29 | 23100 |
| Māori | F | 30-34 | 21900 |
| Māori | F | 35-39 | 22300 |
| Māori | F | 40-44 | 21800 |
| Māori | F | 45-49 | 20500 |
| Māori | F | 50-54 | 17700 |
| Māori | F | 55-59 | 13100 |
| Māori | F | 60-64 | 10100 |
| Māori | F | 65-69 | 6600 |
| Māori | F | 70-74 | 5100 |
| Māori | F | 75-79 | 3100 |
| Māori | F | 80-84 | 1700 |
| Māori | F | 85-89 | 700 |
| Māori | F | 90-94 | 225 |
| Māori | F | 94-99 | 52.5 |
| Māori | F | 100-104 | 18.75 |
| Māori | F | 105-109 | 3.75 |

Supplementary Table 3: Mortality Rate ratios for Māori Females

| **Ethnicity** | **Sex** | **Age** | **Rate ratio (dep5/dep1)** | **Rate ratio (dep4/dep1)** | **Rate ratio (dep3/dep1)** | **Rate ratio (dep2/dep1)** | **Rate ratio (dep1/dep1)** |
| --- | --- | --- | --- | --- | --- | --- | --- |
| Māori | F | 0-24 | 1 | 1 | 1 | 1 | 1 |
| Māori | F | 25-29 | 1.584513 | 1.438385 | 1.292256 | 1.146128 | 1 |
| Māori | F | 30-34 | 1.595621 | 1.446715 | 1.29781 | 1.148905 | 1 |
| Māori | F | 35-39 | 1.612428 | 1.459321 | 1.306214 | 1.153107 | 1 |
| Māori | F | 40-44 | 1.635115 | 1.476336 | 1.317557 | 1.158779 | 1 |
| Māori | F | 45-49 | 1.663922 | 1.497942 | 1.331961 | 1.165981 | 1 |
| Māori | F | 50-54 | 1.699161 | 1.524371 | 1.349581 | 1.17479 | 1 |
| Māori | F | 55-59 | 1.741219 | 1.555914 | 1.37061 | 1.185305 | 1 |
| Māori | F | 60-64 | 1.581209 | 1.435907 | 1.290605 | 1.145302 | 1 |
| Māori | F | 65-69 | 1.461618 | 1.346214 | 1.230809 | 1.115405 | 1 |
| Māori | F | 70-74 | 1.343452 | 1.257589 | 1.171726 | 1.085863 | 1 |
| Māori | F | 75-79 | 1.227879 | 1.170909 | 1.113939 | 1.05697 | 1 |
| Māori | F | 80-84 | 1.115918 | 1.086938 | 1.057959 | 1.028979 | 1 |
| Māori | F | 85-89 | 1.008447 | 1.006335 | 1.004223 | 1.002112 | 1 |
| Māori | F | 90+ | 0.886413 | 0.91481 | 0.943207 | 0.971603 | 1 |

Supplementary Table 4: Morbidity Risk ratios for Māori Females

| **Ethnicity** | **Sex** | | **Age** | | **Risk ratio (dep5/dep1)** | | **Risk ratio (dep4/dep1)** | | **Risk ratio (dep3/dep1)** | | **Risk ratio (dep2/dep1)** | | **Risk ratio (dep1/dep1)** | |
| --- | --- | --- | --- | --- | --- | --- | --- | --- | --- | --- | --- | --- | --- | --- |
| Māori | | F | | 0-24 | | 1 | | 1 | | 1 | | 1 | | 1 |
| Māori | | F | | 25-29 | | 1.233833 | | 1.175374 | | 1.116916 | | 1.058458 | | 1 |
| Māori | | F | | 30-34 | | 1.187804 | | 1.140853 | | 1.093902 | | 1.046951 | | 1 |
| Māori | | F | | 35-39 | | 1.21267 | | 1.159503 | | 1.106335 | | 1.053168 | | 1 |
| Māori | | F | | 40-44 | | 1.175167 | | 1.131375 | | 1.087583 | | 1.043792 | | 1 |
| Māori | | F | | 45-49 | | 1.129293 | | 1.09697 | | 1.064647 | | 1.032323 | | 1 |
| Māori | | F | | 50-54 | | 1.157673 | | 1.118255 | | 1.078836 | | 1.039418 | | 1 |
| Māori | | F | | 55-59 | | 1.143996 | | 1.107997 | | 1.071998 | | 1.035999 | | 1 |
| Māori | | F | | 60-64 | | 1.127272 | | 1.095454 | | 1.063636 | | 1.031818 | | 1 |
| Māori | | F | | 65-69 | | 1.080785 | | 1.060588 | | 1.040392 | | 1.020196 | | 1 |
| Māori | | F | | 70-74 | | 1.064367 | | 1.048275 | | 1.032184 | | 1.016092 | | 1 |
| Māori | | F | | 75-79 | | 1.050946 | | 1.03821 | | 1.025473 | | 1.012737 | | 1 |
| Māori | | F | | 80-84 | | 1.042091 | | 1.031568 | | 1.021045 | | 1.010523 | | 1 |
| Māori | | F | | 85-89 | | 1.006856 | | 1.005142 | | 1.003428 | | 1.001714 | | 1 |
| Māori | | F | | 90+ | | 0.848308 | | 0.886231 | | 0.924154 | | 0.962077 | | 1 |

Supplementary Table 5: CHD rates for Māori Females

| **Ethnicity** | **Sex** | **Age** | **Prevalence** | **Incidence** | **Fatality** | **Disability Rate** |
| --- | --- | --- | --- | --- | --- | --- |
| Māori | F | 0 | 0 | 0 | 0 | 0.096268 |
| Māori | F | 1 | 0 | 0 | 0 | 0.096268 |
| Māori | F | 2 | 0 | 0 | 0 | 0.096268 |
| Māori | F | 3 | 0 | 0 | 0 | 0.096268 |
| Māori | F | 4 | 0 | 0 | 0 | 0.096268 |
| Māori | F | 5 | 0 | 0 | 0 | 0.096268 |
| Māori | F | 6 | 0 | 0 | 0 | 0.096268 |
| Māori | F | 7 | 0 | 0 | 0 | 0.096268 |
| Māori | F | 8 | 0 | 0 | 0 | 0.096268 |
| Māori | F | 9 | 0 | 0 | 0 | 0.096268 |
| Māori | F | 10 | 0 | 0 | 0 | 0.096268 |
| Māori | F | 11 | 0 | 0 | 0 | 0.096268 |
| Māori | F | 12 | 0 | 0 | 0 | 0.096268 |
| Māori | F | 13 | 0 | 0 | 0.005431 | 0.096268 |
| Māori | F | 14 | 0 | 0 | 0.016293 | 0.096268 |
| Māori | F | 15 | 0 | 0 | 0.032585 | 0.096268 |
| Māori | F | 16 | 0 | 0 | 0.054309 | 0.096268 |
| Māori | F | 17 | 0 | 0 | 0.081463 | 0.096268 |
| Māori | F | 18 | 0 | 0 | 0.114048 | 0.096268 |
| Māori | F | 19 | 8.6531E-05 | 4.65E-05 | 0.142915 | 0.096268 |
| Māori | F | 20 | 0.00012766 | 6.9E-05 | 0.162633 | 0.096268 |
| Māori | F | 21 | 0.00018458 | 9.78E-05 | 0.173202 | 0.096268 |
| Māori | F | 22 | 0.0002609 | 0.000133 | 0.174623 | 0.096268 |
| Māori | F | 23 | 0.0003611 | 0.000174 | 0.166894 | 0.096268 |
| Māori | F | 24 | 0.00049148 | 0.000221 | 0.150016 | 0.096268 |
| Māori | F | 25 | 0.00065551 | 0.000264 | 0.126389 | 0.096268 |
| Māori | F | 26 | 0.00084868 | 0.000295 | 0.105162 | 0.096268 |
| Māori | F | 27 | 0.00106311 | 0.000315 | 0.086334 | 0.096268 |
| Māori | F | 28 | 0.00129157 | 0.000324 | 0.069907 | 0.096268 |
| Māori | F | 29 | 0.00152665 | 0.000322 | 0.055878 | 0.096268 |
| Māori | F | 30 | 0.00176014 | 0.000309 | 0.04425 | 0.096268 |
| Māori | F | 31 | 0.00198558 | 0.000294 | 0.036755 | 0.096268 |
| Māori | F | 32 | 0.00220548 | 0.000288 | 0.030996 | 0.096268 |
| Māori | F | 33 | 0.0024283 | 0.000292 | 0.02697 | 0.096268 |
| Māori | F | 34 | 0.00266111 | 0.000306 | 0.024679 | 0.096268 |
| Māori | F | 35 | 0.00290969 | 0.000329 | 0.024122 | 0.096268 |
| Māori | F | 36 | 0.00317849 | 0.000361 | 0.0253 | 0.096268 |
| Māori | F | 37 | 0.00348683 | 0.000434 | 0.027466 | 0.096268 |
| Māori | F | 38 | 0.00386711 | 0.000537 | 0.028885 | 0.096268 |
| Māori | F | 39 | 0.00434901 | 0.000671 | 0.029557 | 0.096268 |
| Māori | F | 40 | 0.004962 | 0.000836 | 0.029484 | 0.096268 |
| Māori | F | 41 | 0.00573568 | 0.001031 | 0.028663 | 0.096268 |
| Māori | F | 42 | 0.00670044 | 0.001257 | 0.027096 | 0.096268 |
| Māori | F | 43 | 0.00789852 | 0.001537 | 0.02504 | 0.096268 |
| Māori | F | 44 | 0.00936581 | 0.001839 | 0.02324 | 0.096268 |
| Māori | F | 45 | 0.01112029 | 0.002165 | 0.021698 | 0.11249 |
| Māori | F | 46 | 0.01317935 | 0.002514 | 0.020412 | 0.11249 |
| Māori | F | 47 | 0.01555942 | 0.002887 | 0.019383 | 0.11249 |
| Māori | F | 48 | 0.01827551 | 0.003282 | 0.018612 | 0.11249 |
| Māori | F | 49 | 0.02132469 | 0.003667 | 0.018112 | 0.11249 |
| Māori | F | 50 | 0.02467794 | 0.00402 | 0.017628 | 0.11249 |
| Māori | F | 51 | 0.02829785 | 0.004339 | 0.01716 | 0.11249 |
| Māori | F | 52 | 0.03214814 | 0.004625 | 0.016707 | 0.11249 |
| Māori | F | 53 | 0.0361936 | 0.004879 | 0.016269 | 0.11249 |
| Māori | F | 54 | 0.04040007 | 0.005099 | 0.015847 | 0.11249 |
| Māori | F | 55 | 0.04478414 | 0.005407 | 0.015777 | 0.123975 |
| Māori | F | 56 | 0.04942521 | 0.005835 | 0.016043 | 0.123975 |
| Māori | F | 57 | 0.05441109 | 0.006384 | 0.016646 | 0.123975 |
| Māori | F | 58 | 0.05982106 | 0.007053 | 0.017586 | 0.123975 |
| Māori | F | 59 | 0.06572488 | 0.007842 | 0.018862 | 0.123975 |
| Māori | F | 60 | 0.07218178 | 0.008751 | 0.020475 | 0.123975 |
| Māori | F | 61 | 0.07921749 | 0.009712 | 0.02216 | 0.123975 |
| Māori | F | 62 | 0.08678621 | 0.010604 | 0.023583 | 0.123975 |
| Māori | F | 63 | 0.09480191 | 0.011427 | 0.024743 | 0.123975 |
| Māori | F | 64 | 0.10318604 | 0.012181 | 0.025639 | 0.123975 |
| Māori | F | 65 | 0.11186833 | 0.012867 | 0.026272 | 0.109541 |
| Māori | F | 66 | 0.12078727 | 0.013483 | 0.026642 | 0.109541 |
| Māori | F | 67 | 0.12988245 | 0.01403 | 0.026881 | 0.109541 |
| Māori | F | 68 | 0.13910704 | 0.014576 | 0.027255 | 0.109541 |
| Māori | F | 69 | 0.14843047 | 0.015121 | 0.027761 | 0.109541 |
| Māori | F | 70 | 0.15782072 | 0.015666 | 0.028402 | 0.109541 |
| Māori | F | 71 | 0.1672445 | 0.01621 | 0.029175 | 0.109541 |
| Māori | F | 72 | 0.17666751 | 0.016753 | 0.030082 | 0.109541 |
| Māori | F | 73 | 0.18608633 | 0.017485 | 0.031713 | 0.109541 |
| Māori | F | 74 | 0.19553112 | 0.018407 | 0.033934 | 0.109541 |
| Māori | F | 75 | 0.2050256 | 0.01952 | 0.036745 | 0.100756 |
| Māori | F | 76 | 0.21457837 | 0.020823 | 0.040147 | 0.100756 |
| Māori | F | 77 | 0.22418386 | 0.022316 | 0.04414 | 0.100756 |
| Māori | F | 78 | 0.23382357 | 0.023999 | 0.048723 | 0.100756 |
| Māori | F | 79 | 0.24337891 | 0.025619 | 0.053848 | 0.100756 |
| Māori | F | 80 | 0.25262657 | 0.026986 | 0.058927 | 0.100756 |
| Māori | F | 81 | 0.26134547 | 0.028099 | 0.063958 | 0.100756 |
| Māori | F | 82 | 0.26934014 | 0.028959 | 0.068941 | 0.100756 |
| Māori | F | 83 | 0.27643974 | 0.029566 | 0.073878 | 0.100756 |
| Māori | F | 84 | 0.28249618 | 0.029919 | 0.078766 | 0.100756 |
| Māori | F | 85 | 0.28723392 | 0.029987 | 0.085003 | 0.089604 |
| Māori | F | 86 | 0.29033412 | 0.030022 | 0.092634 | 0.089604 |
| Māori | F | 87 | 0.29158889 | 0.030024 | 0.10166 | 0.089604 |
| Māori | F | 88 | 0.29081797 | 0.029995 | 0.112082 | 0.089604 |
| Māori | F | 89 | 0.28787713 | 0.029932 | 0.123898 | 0.089604 |
| Māori | F | 90 | 0.28266789 | 0.029838 | 0.137109 | 0.089604 |
| Māori | F | 91 | 0.27504134 | 0.030028 | 0.154038 | 0.089604 |
| Māori | F | 92 | 0.26497195 | 0.030535 | 0.17329 | 0.089604 |
| Māori | F | 93 | 0.25270519 | 0.03136 | 0.194863 | 0.089604 |
| Māori | F | 94 | 0.23860577 | 0.032503 | 0.21876 | 0.089604 |
| Māori | F | 95 | 0.2231577 | 0.033962 | 0.244978 | 0.089604 |
| Māori | F | 96 | 0.20694661 | 0.035739 | 0.27352 | 0.089604 |
| Māori | F | 97 | 0.19080747 | 0.037535 | 0.299974 | 0.089604 |
| Māori | F | 98 | 0.17544101 | 0.039509 | 0.329074 | 0.089604 |
| Māori | F | 99 | 0.16288367 | 0.041051 | 0.35599 | 0.089604 |
| Māori | F | 100 | 0.16288367 | 0.041051 | 0.35599 | 0.089604 |
| Māori | F | 101 | 0.16288367 | 0.041051 | 0.35599 | 0.089604 |
| Māori | F | 102 | 0.16288367 | 0.041051 | 0.35599 | 0.089604 |
| Māori | F | 103 | 0.16288367 | 0.041051 | 0.35599 | 0.089604 |
| Māori | F | 104 | 0.16288367 | 0.041051 | 0.35599 | 0.089604 |
| Māori | F | 105 | 0.16288367 | 0.041051 | 0.35599 | 0.089604 |
| Māori | F | 106 | 0.16288367 | 0.041051 | 0.35599 | 0.089604 |
| Māori | F | 107 | 0.16288367 | 0.041051 | 0.35599 | 0.089604 |
| Māori | F | 108 | 0.16288367 | 0.041051 | 0.35599 | 0.089604 |
| Māori | F | 109 | 0.16288367 | 0.041051 | 0.35599 | 0.089604 |
| Māori | F | 110 | 0.16288367 | 0.041051 | 0.35599 | 0.089604 |

Supplementary Table 6: CHD Incidence rate ratios for Māori Females

The data in supplementary tables 6, 8, 10, and 12 were based on extracts from the following NZ national health collections: National Health Index (NHI), Primary Health Organisation (PHO) Enrolment collection, Laboratory Claims, Pharmaceutical Claims, National Minimum Dataset (NMDS) - publicly funded hospitalizations only, Cancer Registry, Mortality Collection, National Non-Admitted Patient Collection, General Medical Subsidy (GMS) Claims.

**Health Service user population, and disease indications**

We created a health service user population for the 2011/12 financial year based on recorded contact and residence status in the above collections. Amongst this group of people, we used the algorithms described below to flag whether or not an individual had shown an indication of CHD or stroke in 2011/12:

**CHD:** People with a publicly-funded hospital discharge with any (primary or otherwise) diagnosis of ICD-10-AM codes I20-I25 on or before the specified financial year who were alive for at least part of the year, or with an underlying cause of death recorded as one of these ICD-10-codes within the specified financial year, or with one of the following publicly-funded hospital procedures during the specified financial year:

(ICD-10-AM: 3530400, 3530500, 3531000, 3531001, 3531002, 3849700, 3849701, 3849702, 3849703, 3849704, 3849705, 3849706, 3849707, 3850000, 3850001, 3850002, 3850003, 3850004, 3850300, 3850301, 3850302, 3850303, 3850304, 3863700, 9020100, 9020101, 9020102, 9020103)

or with two or more dispensings of one of the following pharmaceuticals :

(Glyceryl trinitrate 1577, Isosorbide Dinitrate 2377 , Isosorbide mononitrate 2836, Nicorandil 1272, Perhexiline maleate 1949) in the specified financial year

**Stroke**: People with a publicly-funded hospital discharge with any (primary or otherwise) diagnosis of ICD-10-AM codes G45-G46, I60-I67 on or before the specified financial year who were alive for at least part of the year, or with an underlying cause of death recorded as one of these ICD-10-codes, during the specified financial year

**NZ Deprivation quintile**

We created a variable to flag NZ Deprivation Index quintile (as there is no standard NZDep variable as part of these collections) based on the following method:

We extracted all domicile codes for each person for the years 2004/05 to 2014/15, along with relevant dates, from the following collections: NMDS (event end date), NHI (last updated date), Cancer registrations (diagnosis date), Mortality (date of death), NNPAC (date of service), PHO (last consultation date). Note that one person can have many domicile codes over a year.

The domicile codes were mapped to census area units, which in turn were mapped to NZ Dep quintiles, first using 2013 mappings (and then if they didn't work, 2006 mappings). Where an individual in the health service user population had more than one NZDep quintile value for a particular financial year, we assigned the quintile value closest to the end of the financial year. Where an individual did not have an NZDep quintile for a particular financial year, we used an NZDep quintile from the closest financial year (looking back first). A maximum five year range was used (that is, two years either side of the particular year). Finally, the NZDep quintile based on the domicile code from the NHI – which represented the NZDep from the last recorded (i.e. most recent) domicile code was added on to fill any remaining gaps.

**Incidence**

Incidence was measured as counts of people with their first indication of CHD or Stroke in 2011/12 divided by person time at risk (based on the health service use population). When incidence was calculated by age, sex, ethnic, and deprivation quintile group, the numbers were very small and patterns in rates were unstable. Predicted rates were calculated using Poisson regression (or negative binomial if overdispersed).

**Prevalence**

Prevalence was calculated as a proportion with number of cases (of CHD or stroke) divided by the health service user population. As for the incidence measure, rates were unstable when broken down by the four demographic variables, so logistic regression was used to calculate predicted rates.

| **Ethnicity** | **Sex** | **Age** | **Rate ratio (dep5/dep1)** | **Rate ratio (dep4/dep1)** | **Rate ratio (dep3/dep1)** | **Rate ratio (dep2/dep1)** | **Rate ratio (dep1/dep1)** |
| --- | --- | --- | --- | --- | --- | --- | --- |
| Māori | F | 0-24 | 1 | 1 | 1 | 1 | 1 |
| Māori | F | 25-29 | 2.508118 | 2.131088 | 1.754059 | 1.377029 | 1 |
| Māori | F | 30-34 | 2.447497 | 2.085623 | 1.723748 | 1.361874 | 1 |
| Māori | F | 35-39 | 2.359299 | 2.019474 | 1.67965 | 1.339825 | 1 |
| Māori | F | 40-44 | 2.246627 | 1.93497 | 1.623314 | 1.311657 | 1 |
| Māori | F | 45-49 | 2.113324 | 1.834993 | 1.556662 | 1.278331 | 1 |
| Māori | F | 50-54 | 1.963758 | 1.722819 | 1.481879 | 1.24094 | 1 |
| Māori | F | 55-59 | 1.80259 | 1.601942 | 1.401295 | 1.200647 | 1 |
| Māori | F | 60-64 | 1.717948 | 1.538461 | 1.358974 | 1.179487 | 1 |
| Māori | F | 65-69 | 1.533512 | 1.400134 | 1.266756 | 1.133378 | 1 |
| Māori | F | 70-74 | 1.384246 | 1.288185 | 1.192123 | 1.096062 | 1 |
| Māori | F | 75-79 | 1.26354 | 1.197655 | 1.13177 | 1.065885 | 1 |
| Māori | F | 80-84 | 1.166309 | 1.124732 | 1.083154 | 1.041577 | 1 |
| Māori | F | 85-89 | 1.088648 | 1.066486 | 1.044324 | 1.022162 | 1 |
| Māori | F | 90+ | 1.017131 | 1.012848 | 1.008565 | 1.004283 | 1 |

Supplementary Table 7: CHD Fatality rate ratios for Māori Females

| **Ethnicity** | **Sex** | **Age** | **Rate ratio (dep5/dep1)** | **Rate ratio (dep4/dep1)** | **Rate ratio (dep3/dep1)** | **Rate ratio (dep2/dep1)** | **Rate ratio (dep1/dep1)** |
| --- | --- | --- | --- | --- | --- | --- | --- |
| Māori | F | 0-24 | 1 | 1 | 1 | 1 | 1 |
| Māori | F | 25-29 | 1.48253 | 1.361897 | 1.241265 | 1.120632 | 1 |
| Māori | F | 30-34 | 1.456976 | 1.342732 | 1.228488 | 1.114244 | 1 |
| Māori | F | 35-39 | 1.419471 | 1.314603 | 1.209735 | 1.104868 | 1 |
| Māori | F | 40-44 | 1.370961 | 1.27822 | 1.18548 | 1.09274 | 1 |
| Māori | F | 45-49 | 1.312648 | 1.984486 | 1.156324 | 1.078162 | 1 |
| Māori | F | 50-54 | 1.245937 | 1.934453 | 1.122969 | 1.061484 | 1 |
| Māori | F | 55-59 | 1.172381 | 1.879286 | 1.086191 | 1.043095 | 1 |
| Māori | F | 60-64 | 1.187543 | 1.890657 | 1.093771 | 1.046886 | 1 |
| Māori | F | 65-69 | 1.189493 | 1.892119 | 1.094746 | 1.047373 | 1 |
| Māori | F | 70-74 | 1.167084 | 1.125313 | 1.083542 | 1.041771 | 1 |
| Māori | F | 75-79 | 1.121684 | 1.091263 | 1.060842 | 1.030421 | 1 |
| Māori | F | 80-84 | 1.056009 | 1.042007 | 1.028005 | 1.014002 | 1 |
| Māori | F | 85-89 | 0.973852 | 0.980389 | 0.986926 | 0.993463 | 1 |
| Māori | F | 90+ | 0.859885 | 0.894913 | 0.929942 | 0.964971 | 1 |

Supplementary Table 8: CHD Prevalence ratios for Māori Females

| **Ethnicity** | **Sex** | **Age** | **Rate ratio (dep5/dep1)** | **Rate ratio (dep4/dep1)** | **Rate ratio (dep3/dep1)** | **Rate ratio (dep2/dep1)** | **Rate ratio (dep1/dep1)** |
| --- | --- | --- | --- | --- | --- | --- | --- |
| Māori | F | 0-24 | 1 | 1 | 1 | 1 | 1 |
| Māori | F | 25-29 | 2.137763 | 1.853322 | 1.568881 | 1.284441 | 1 |
| Māori | F | 30-34 | 2.127309 | 1.845482 | 1.563655 | 1.281827 | 1 |
| Māori | F | 35-39 | 2.102494 | 1.826871 | 1.551247 | 1.275624 | 1 |
| Māori | F | 40-44 | 2.063819 | 1.797864 | 1.53191 | 1.265955 | 1 |
| Māori | F | 45-49 | 2.012061 | 1.759046 | 1.50603 | 1.253015 | 1 |
| Māori | F | 50-54 | 1.948246 | 1.711185 | 1.474123 | 1.237062 | 1 |
| Māori | F | 55-59 | 1.873609 | 1.655207 | 1.436805 | 1.218402 | 1 |
| Māori | F | 60-64 | 1.697884 | 1.523413 | 1.348942 | 1.174471 | 1 |
| Māori | F | 65-69 | 1.483933 | 1.36295 | 1.241967 | 1.120983 | 1 |
| Māori | F | 70-74 | 1.323865 | 1.242899 | 1.161933 | 1.080966 | 1 |
| Māori | F | 75-79 | 1.205583 | 1.154187 | 1.102791 | 1.051396 | 1 |
| Māori | F | 80-84 | 1.120657 | 1.090493 | 1.060329 | 1.030164 | 1 |
| Māori | F | 85-89 | 1.063342 | 1.047506 | 1.031671 | 1.015835 | 1 |
| Māori | F | 90+ | 1.025867 | 1.019401 | 1.012934 | 1.006467 | 1 |

Supplementary Table 9: Stroke rates for Māori Females

| **Ethnicity** | **Sex** | **Age** | **Prevalence** | **Incidence** | **Fatality** | **Disability Rate** |
| --- | --- | --- | --- | --- | --- | --- |
| Māori | F | 0 | 0 | 0 | 0 | 0.431037 |
| Māori | F | 1 | 0 | 0 | 0 | 0.431037 |
| Māori | F | 2 | 0 | 0 | 0 | 0.431037 |
| Māori | F | 3 | 0 | 0 | 0 | 0.431037 |
| Māori | F | 4 | 0 | 0 | 0 | 0.431037 |
| Māori | F | 5 | 0 | 0 | 0 | 0.431037 |
| Māori | F | 6 | 0 | 0 | 0 | 0.431037 |
| Māori | F | 7 | 0 | 0 | 0 | 0.431037 |
| Māori | F | 8 | 0 | 0 | 0 | 0.431037 |
| Māori | F | 9 | 0 | 0 | 0 | 0.431037 |
| Māori | F | 10 | 0 | 0 | 0 | 0.431037 |
| Māori | F | 11 | 0 | 0 | 0 | 0.431037 |
| Māori | F | 12 | 0 | 0 | 0 | 0.431037 |
| Māori | F | 13 | 0 | 0 | 0 | 0.431037 |
| Māori | F | 14 | 0 | 0 | 0 | 0.431037 |
| Māori | F | 15 | 0 | 0 | 0 | 0.431037 |
| Māori | F | 16 | 0 | 0 | 0 | 0.431037 |
| Māori | F | 17 | 0 | 0 | 0 | 0.431037 |
| Māori | F | 18 | 0 | 0 | 0 | 0.431037 |
| Māori | F | 19 | 0 | 0 | 0 | 0.431037 |
| Māori | F | 20 | 0 | 0 | 0 | 0.431037 |
| Māori | F | 21 | 0 | 0 | 0 | 0.431037 |
| Māori | F | 22 | 0 | 0 | 0 | 0.431037 |
| Māori | F | 23 | 0 | 0 | 0 | 0.431037 |
| Māori | F | 24 | 0 | 0 | 0 | 0.431037 |
| Māori | F | 25 | 5.48E-07 | 1.12E-06 | 0.047619 | 0.431037 |
| Māori | F | 26 | 2.59E-06 | 3.37E-06 | 0.142857 | 0.431037 |
| Māori | F | 27 | 6.5E-06 | 6.73E-06 | 0.285714 | 0.431037 |
| Māori | F | 28 | 1.17E-05 | 1.12E-05 | 0.47619 | 0.431037 |
| Māori | F | 29 | 1.68E-05 | 1.68E-05 | 0.714286 | 0.431037 |
| Māori | F | 30 | 2.05E-05 | 2.36E-05 | 1 | 0.431037 |
| Māori | F | 31 | 2.3E-05 | 3.2E-05 | 1.285714 | 0.431037 |
| Māori | F | 32 | 2.52E-05 | 4.1E-05 | 1.52381 | 0.431037 |
| Māori | F | 33 | 2.76E-05 | 5.07E-05 | 1.714286 | 0.431037 |
| Māori | F | 34 | 3.06E-05 | 6.09E-05 | 1.857143 | 0.431037 |
| Māori | F | 35 | 3.41E-05 | 7.17E-05 | 1.952381 | 0.431037 |
| Māori | F | 36 | 3.84E-05 | 8.31E-05 | 2 | 0.431037 |
| Māori | F | 37 | 5.03E-05 | 0.000123 | 1.95321 | 0.431037 |
| Māori | F | 38 | 7.8E-05 | 0.000191 | 1.859631 | 0.431037 |
| Māori | F | 39 | 0.000125 | 0.000287 | 1.719261 | 0.431037 |
| Māori | F | 40 | 0.000199 | 0.000411 | 1.532102 | 0.431037 |
| Māori | F | 41 | 0.000313 | 0.000564 | 1.298153 | 0.431037 |
| Māori | F | 42 | 0.000493 | 0.000744 | 1.017414 | 0.431037 |
| Māori | F | 43 | 0.000781 | 0.000943 | 0.736539 | 0.431037 |
| Māori | F | 44 | 0.001213 | 0.001133 | 0.502318 | 0.431037 |
| Māori | F | 45 | 0.001834 | 0.001315 | 0.314749 | 0.137442 |
| Māori | F | 46 | 0.002705 | 0.001487 | 0.173835 | 0.137442 |
| Māori | F | 47 | 0.003879 | 0.001651 | 0.079573 | 0.137442 |
| Māori | F | 48 | 0.005359 | 0.001805 | 0.031965 | 0.137442 |
| Māori | F | 49 | 0.007026 | 0.001939 | 0.031062 | 0.137442 |
| Māori | F | 50 | 0.00877 | 0.002061 | 0.030209 | 0.137442 |
| Māori | F | 51 | 0.010581 | 0.002172 | 0.029406 | 0.137442 |
| Māori | F | 52 | 0.012447 | 0.002272 | 0.028654 | 0.137442 |
| Māori | F | 53 | 0.014358 | 0.00236 | 0.027953 | 0.137442 |
| Māori | F | 54 | 0.016302 | 0.002436 | 0.027303 | 0.137442 |
| Māori | F | 55 | 0.018276 | 0.002517 | 0.026902 | 0.129704 |
| Māori | F | 56 | 0.020284 | 0.002613 | 0.0267 | 0.129704 |
| Māori | F | 57 | 0.022339 | 0.002725 | 0.026697 | 0.129704 |
| Māori | F | 58 | 0.02445 | 0.002853 | 0.026893 | 0.129704 |
| Māori | F | 59 | 0.026625 | 0.002996 | 0.027287 | 0.129704 |
| Māori | F | 60 | 0.028869 | 0.003155 | 0.02788 | 0.129704 |
| Māori | F | 61 | 0.031193 | 0.003338 | 0.02869 | 0.129704 |
| Māori | F | 62 | 0.033602 | 0.00353 | 0.029518 | 0.129704 |
| Māori | F | 63 | 0.036098 | 0.003731 | 0.030364 | 0.129704 |
| Māori | F | 64 | 0.03868 | 0.003941 | 0.031228 | 0.129704 |
| Māori | F | 65 | 0.04135 | 0.004159 | 0.03211 | 0.163902 |
| Māori | F | 66 | 0.044107 | 0.004387 | 0.03301 | 0.163902 |
| Māori | F | 67 | 0.046947 | 0.004626 | 0.034107 | 0.163902 |
| Māori | F | 68 | 0.049861 | 0.004867 | 0.035384 | 0.163902 |
| Māori | F | 69 | 0.052832 | 0.005111 | 0.036841 | 0.163902 |
| Māori | F | 70 | 0.055843 | 0.005357 | 0.038478 | 0.163902 |
| Māori | F | 71 | 0.058878 | 0.005606 | 0.040295 | 0.163902 |
| Māori | F | 72 | 0.061917 | 0.005858 | 0.042291 | 0.163902 |
| Māori | F | 73 | 0.064941 | 0.006115 | 0.044522 | 0.163902 |
| Māori | F | 74 | 0.067936 | 0.006376 | 0.046807 | 0.163902 |
| Māori | F | 75 | 0.070889 | 0.00664 | 0.049147 | 0.258457 |
| Māori | F | 76 | 0.07379 | 0.006908 | 0.051542 | 0.258457 |
| Māori | F | 77 | 0.076629 | 0.007179 | 0.053991 | 0.258457 |
| Māori | F | 78 | 0.079396 | 0.007453 | 0.056495 | 0.258457 |
| Māori | F | 79 | 0.082061 | 0.007898 | 0.061678 | 0.258457 |
| Māori | F | 80 | 0.084567 | 0.008512 | 0.069485 | 0.258457 |
| Māori | F | 81 | 0.086845 | 0.009293 | 0.079916 | 0.258457 |
| Māori | F | 82 | 0.088821 | 0.010243 | 0.092971 | 0.258457 |
| Māori | F | 83 | 0.090421 | 0.01136 | 0.10865 | 0.258457 |
| Māori | F | 84 | 0.091581 | 0.012646 | 0.126953 | 0.258457 |
| Māori | F | 85 | 0.092002 | 0.014028 | 0.153503 | 0.558867 |
| Māori | F | 86 | 0.091237 | 0.015338 | 0.185677 | 0.558867 |
| Māori | F | 87 | 0.089015 | 0.016576 | 0.223474 | 0.558867 |
| Māori | F | 88 | 0.085244 | 0.017742 | 0.266895 | 0.558867 |
| Māori | F | 89 | 0.080037 | 0.018837 | 0.315939 | 0.558867 |
| Māori | F | 90 | 0.073694 | 0.019861 | 0.370606 | 0.558867 |
| Māori | F | 91 | 0.066711 | 0.020792 | 0.428389 | 0.558867 |
| Māori | F | 92 | 0.059802 | 0.021703 | 0.483665 | 0.558867 |
| Māori | F | 93 | 0.053618 | 0.022593 | 0.536433 | 0.558867 |
| Māori | F | 94 | 0.048464 | 0.023463 | 0.586694 | 0.558867 |
| Māori | F | 95 | 0.044383 | 0.024313 | 0.634447 | 0.558867 |
| Māori | F | 96 | 0.041262 | 0.025143 | 0.679693 | 0.558867 |
| Māori | F | 97 | 0.038963 | 0.025836 | 0.716325 | 0.558867 |
| Māori | F | 98 | 0.037271 | 0.026599 | 0.756621 | 0.558867 |
| Māori | F | 99 | 0.036039 | 0.02712 | 0.793784 | 0.558867 |
| Māori | F | 100 | 0.036039 | 0.02712 | 0.793784 | 0.558867 |
| Māori | F | 101 | 0.036039 | 0.02712 | 0.793784 | 0.558867 |
| Māori | F | 102 | 0.036039 | 0.02712 | 0.793784 | 0.558867 |
| Māori | F | 103 | 0.036039 | 0.02712 | 0.793784 | 0.558867 |
| Māori | F | 104 | 0.036039 | 0.02712 | 0.793784 | 0.558867 |
| Māori | F | 105 | 0.036039 | 0.02712 | 0.793784 | 0.558867 |
| Māori | F | 106 | 0.036039 | 0.02712 | 0.793784 | 0.558867 |
| Māori | F | 107 | 0.036039 | 0.02712 | 0.793784 | 0.558867 |
| Māori | F | 108 | 0.036039 | 0.02712 | 0.793784 | 0.558867 |
| Māori | F | 109 | 0.036039 | 0.02712 | 0.793784 | 0.558867 |
| Māori | F | 110 | 0.036039 | 0.02712 | 0.793784 | 0.558867 |

Supplementary Table 10: Stroke Incidence rate ratios for Māori Females

| **Ethnicity** | **Sex** | **Age** | **Rate ratio (dep5/dep1)** | **Rate ratio (dep4/dep1)** | **Rate ratio (dep3/dep1)** | **Rate ratio (dep2/dep1)** | **Rate ratio (dep1/dep1)** |
| --- | --- | --- | --- | --- | --- | --- | --- |
| Māori | F | 0-24 | 1 | 1 | 1 | 1 | 1 |
| Māori | F | 25-29 | 1.573997 | 1.430498 | 1.286999 | 1.143499 | 1 |
| Māori | F | 30-34 | 1.597383 | 1.448037 | 1.298691 | 1.149346 | 1 |
| Māori | F | 35-39 | 1.633113 | 1.474835 | 1.316557 | 1.158278 | 1 |
| Māori | F | 40-44 | 1.682 | 1.5115 | 1.341 | 1.1705 | 1 |
| Māori | F | 45-49 | 1.745172 | 1.558879 | 1.372586 | 1.186293 | 1 |
| Māori | F | 50-54 | 1.824119 | 1.618089 | 1.412059 | 1.20603 | 1 |
| Māori | F | 55-59 | 1.920749 | 1.690562 | 1.460375 | 1.230187 | 1 |
| Māori | F | 60-64 | 1.663498 | 1.497623 | 1.331749 | 1.165874 | 1 |
| Māori | F | 65-69 | 1.438686 | 1.329014 | 1.219343 | 1.109671 | 1 |
| Māori | F | 70-74 | 1.273003 | 1.204752 | 1.136501 | 1.068251 | 1 |
| Māori | F | 75-79 | 1.152425 | 1.114319 | 1.076212 | 1.038106 | 1 |
| Māori | F | 80-84 | 1.067372 | 1.050529 | 1.033686 | 1.016843 | 1 |
| Māori | F | 85-89 | 1.011437 | 1.008578 | 1.005719 | 1.002859 | 1 |
| Māori | F | 90+ | 0.977196 | 0.982897 | 0.988598 | 0.994299 | 1 |

Supplementary Table 11: Stroke Fatality rate ratios for Māori Females

| **Ethnicity** | **Sex** | **Age** | **Rate ratio (dep5/dep1)** | **Rate ratio (dep4/dep1)** | **Rate ratio (dep3/dep1)** | **Rate ratio (dep2/dep1)** | **Rate ratio (dep1/dep1)** |
| --- | --- | --- | --- | --- | --- | --- | --- |
| Māori | F | 0-24 | 1 | 1 | 1 | 1 | 1 |
| Māori | F | 25-29 | 1.161292 | 1.120969 | 1.080646 | 1.040323 | 1 |
| Māori | F | 30-34 | 1.176665 | 1.132499 | 1.088332 | 1.044166 | 1 |
| Māori | F | 35-39 | 1.200108 | 1.150081 | 1.100054 | 1.050027 | 1 |
| Māori | F | 40-44 | 1.232093 | 1.174069 | 1.116046 | 1.058023 | 1 |
| Māori | F | 45-49 | 1.273275 | 1.954957 | 1.136638 | 1.068319 | 1 |
| Māori | F | 50-54 | 1.324515 | 1.993387 | 1.162258 | 1.081129 | 1 |
| Māori | F | 55-59 | 1.386907 | 2.04018 | 1.193453 | 1.096727 | 1 |
| Māori | F | 60-64 | 1.294083 | 1.970562 | 1.147041 | 1.073521 | 1 |
| Māori | F | 65-69 | 1.240382 | 1.930287 | 1.120191 | 1.060096 | 1 |
| Māori | F | 70-74 | 1.186802 | 1.140102 | 1.093401 | 1.046701 | 1 |
| Māori | F | 75-79 | 1.133521 | 1.100141 | 1.06676 | 1.03338 | 1 |
| Māori | F | 80-84 | 1.08071 | 1.060532 | 1.040355 | 1.020177 | 1 |
| Māori | F | 85-89 | 1.028531 | 1.021399 | 1.014266 | 1.007133 | 1 |
| Māori | F | 90+ | 0.966962 | 0.975221 | 0.983481 | 0.99174 | 1 |

Supplementary Table 12: Stroke Prevalence ratios for Māori Females

| **Ethnicity** | **Sex** | **Age** | **Rate ratio (dep5/dep1)** | **Rate ratio (dep4/dep1)** | **Rate ratio (dep3/dep1)** | **Rate ratio (dep2/dep1)** | **Rate ratio (dep1/dep1)** |
| --- | --- | --- | --- | --- | --- | --- | --- |
| Māori | F | 0-24 | 1 | 1 | 1 | 1 | 1 |
| Māori | F | 25-29 | 1.549719 | 1.412289 | 1.27486 | 1.13743 | 1 |
| Māori | F | 30-34 | 1.58028 | 1.43521 | 1.29014 | 1.14507 | 1 |
| Māori | F | 35-39 | 1.621962 | 1.466471 | 1.310981 | 1.15549 | 1 |
| Māori | F | 40-44 | 1.675606 | 1.506704 | 1.337803 | 1.168901 | 1 |
| Māori | F | 45-49 | 1.742323 | 1.556742 | 1.371161 | 1.185581 | 1 |
| Māori | F | 50-54 | 1.823519 | 1.617639 | 1.411759 | 1.20588 | 1 |
| Māori | F | 55-59 | 1.920954 | 1.690716 | 1.460477 | 1.230239 | 1 |
| Māori | F | 60-64 | 1.660236 | 1.495177 | 1.330118 | 1.165059 | 1 |
| Māori | F | 65-69 | 1.428493 | 1.321369 | 1.214246 | 1.107123 | 1 |
| Māori | F | 70-74 | 1.261287 | 1.195966 | 1.130644 | 1.065322 | 1 |
| Māori | F | 75-79 | 1.142819 | 1.107114 | 1.07141 | 1.035705 | 1 |
| Māori | F | 80-84 | 1.062598 | 1.046948 | 1.031299 | 1.015649 | 1 |
| Māori | F | 85-89 | 1.013883 | 1.010412 | 1.006941 | 1.003471 | 1 |
| Māori | F | 90+ | 0.991632 | 0.993724 | 0.995816 | 0.997908 | 1 |

Supplementary Table 13: Sodium Daily Intake Proportions for Māori Females (rows sum to 1)

| **Ethnicity** | **Sex** | **Age** | **0-1 gram** | **1-2 gram** | **2-3 gram** | **3-4 gram** | **4-5 gram** | **5-6 gram** | **6-7 gram** | **>7 gram** |
| --- | --- | --- | --- | --- | --- | --- | --- | --- | --- | --- |
| Māori | F | 0-24 | 0.028985507 | 0.217391304 | 0.297101449 | 0.188405797 | 0.115942029 | 0.072463768 | 0.036231884 | 0.043478261 |
| Māori | F | 25-34 | 0.030303 | 0.187879 | 0.218182 | 0.218182 | 0.175758 | 0.090909 | 0.030303 | 0.048485 |
| Māori | F | 35-44 | 0.02963 | 0.2 | 0.266667 | 0.2 | 0.125926 | 0.111111 | 0.02963 | 0.037037 |
| Māori | F | 45-54 | 0.08642 | 0.160494 | 0.333333 | 0.17284 | 0.111111 | 0.049383 | 0.074074 | 0.012346 |
| Māori | F | 55-64 | 0.033333 | 0.283333 | 0.266667 | 0.216667 | 0.1 | 0.05 | 0.016667 | 0.033333 |
| Māori | F | 65-74 | 0.051282 | 0.333333 | 0.333333 | 0.179487 | 0.025641 | 0.051282 | 0.025641 | 0 |
| Māori | F | 75-84 | 0.071429 | 0.428571 | 0.214286 | 0.071429 | 0.071429 | 0.071429 | 0 | 0.071429 |
| Māori | F | 85+ | 0 | 0 | 0.333333 | 0.666667 | 0 | 0 | 0 | 0 |

Supplementary Table 14: Relative Risks for CHD and Stroke per 1g/day Sodium Increase for Māori Females*

| **Ethnicity** | **Sex** | **Age** | **CHD** | **Stroke** |
| --- | --- | --- | --- | --- |
| Māori | F | 0-24 | 1 | 1 |
| Māori | F | 25-29 | 1.04 | 1.05182 |
| Māori | F | 30-34 | 1.05 | 1.06965 |
| Māori | F | 35-39 | 1.057 | 1.08688 |
| Māori | F | 40-44 | 1.063 | 1.10034 |
| Māori | F | 45-49 | 1.073 | 1.10901 |
| Māori | F | 50-54 | 1.082 | 1.11714 |
| Māori | F | 55-59 | 1.088 | 1.12139 |
| Māori | F | 60-64 | 1.091 | 1.12213 |
| Māori | F | 65-69 | 1.093 | 1.11908 |
| Māori | F | 70-74 | 1.084 | 1.10173 |
| Māori | F | 75-79 | 1.074 | 1.08226 |
| Māori | F | 80-84 | 1.058 | 1.04165 |
| Māori | F | 85-89 | 1.058 | 1.04156 |
| Māori | F | 90+ | 1.058 | 1.04152 |

*With a theoretical minimum risk exposure level (TMREL) of 3g sodium/day

Supplementary Table 15: Sodium Daily Intake BAU Mean Exposures for Māori Females

| **Ethnicity** | **Sex** | **0-1 gram** | **1-2 gram** | **2-3 gram** | **3-4 gram** | **4-5 gram** | **5-6 gram** | **6-7 gram** | **>7 gram** |
| --- | --- | --- | --- | --- | --- | --- | --- | --- | --- |
| Māori | F | 0.678652 | 1.574601 | 2.511774 | 3.474541 | 4.441067 | 5.469723 | 6.477527 | 8.845344 |
